# Early-Age Mechanical Characteristics and Microstructure of Concrete Containing Mineral Admixtures under the Environment of Low Humidity and Large Temperature Variation

**DOI:** 10.3390/ma14175085

**Published:** 2021-09-05

**Authors:** Jinjun Guo, Zheng Zhang, Jingjiang Wu, Huikang Wang, Peng Zhang, Kun Wang, Qingxin Meng, Hongyin Xu

**Affiliations:** 1 School of Water Conservancy Engineering, Zhengzhou University, Zhengzhou 450001, China; guojinjun@zzu.edu.cn (J.G.); zheng_zhang1996@163.com (Z.Z.); huikang_wang@163.com (H.W.); zhangpeng@zzu.edu.cn (P.Z.); wangkunli@gs.zzu.edu.cn (K.W.); 2 China Construction Seventh Engineering Division Co., Ltd., Zhengzhou 450001, China; wujingjiang@cscec.com (J.W.); qingxinmeng1995@163.com (Q.M.)

**Keywords:** mechanical characteristics, microstructure, concrete with mineral admixture, low humidity, large temperature variation

## Abstract

The application of concrete containing mineral admixtures was attempted in Northwest China in this study, where the environment has the characteristics of low humidity and large temperature variation. The harsh environment was simulated by using an environmental chamber in the laboratory and four types of concrete were prepared, including ordinary concrete and three kinds of mineral admixture concretes with different contents of fly ash and blast-furnace slag. These concretes were cured in the environmental chamber according to the real curing conditions during construction. The compression strength, fracture properties, SEM images, air-void characteristics, and X-ray diffraction features were researched at the early ages of curing before 28 d. The results showed that the addition of fly ash and slag can improve the compression strength and fracture properties of concrete in the environment of low humidity and large temperature variation. The optimal mixing of mineral admixture was 10% fly ash and 20% slag by replacing the cement in concrete, which can improve the compression strength, initial fracture toughness, unstable fracture toughness, and fracture energy by 23.9%, 25.2%, 45.3%, and 22.6%, respectively, compared to ordinary concrete. Through the analysis of the microstructure of concrete, the addition of fly ash and slag can weaken the negative effects of the harsh environment of low humidity and large temperature variation on concrete microstructure and cement hydration.

## 1. Introduction

In the past three decades, the economy of China developed rapidly. In 2019, China’s GDP reach USD 14.343 trillion, ranking second in the world. However, there is an imbalance between the east and the west of China, and the infrastructure construction is relatively weak in the Northwest China. According to the GDP data of 2019, the total GDP of western region is USD 2.983 trillion, accounting for 20.8% of the national GDP; while the eastern region is USD 7.444 trillion, accounting for 51.9%. In recent years, China has accelerated the pace of infrastructure construction in the Northwest. From 2015 to 2019, China’s road construction investment had increased by 32.6%. In 2019, China completed CNY 10,049.8 billion in road construction investment in the Northwest [1]. However, the local environment in this region is relatively harsh. Firstly, there is a big difference in temperature between day and night, with daily temperature varying by more than 40 °C. Secondly, it is dry and rainless in this region, and the air humidity is low. These harsh environmental characteristics have brought serious challenges to infrastructure construction, especially for concrete materials, which are commonly used in construction.

Existing research has proven that the humidity and temperature in the curing environment have an impact on the mechanical characteristics and microstructure of concrete. When concrete is cured in a dry environment, the water inside the concrete will evaporate to the outside, resulting in the chaos of ion migration inside the concrete and weakening the hydration products in the interface transition zone (ITZ) [2]. AM Soliman [3] investigated the effects of drying conditions on ultra-high-performance concrete (UHPC) at early-ages. The results claimed that the compressive strength of concrete decreased as the curing humidity decreased, and the total shrinkage strain increased. Mi et al. [4] tested the concrete fracture properties using wedge splitting specimens exposed to four different relative humidity values (30%, 50%, 70%, and 98%). The results showed that the fracture energy, effective fracture toughness, and characteristic length increased with the environmental humidity, and the concrete fracture mode also gradually changed from ductile to brittle with curing humidity decreasing and hydration time elapsing. The temperature of the curing environment can also affect the performance of concrete. Wang et al. [5] placed concrete specimens in multiple initial curing temperatures of 40 °C, 50 °C, 60 °C, 70 °C, and 80 °C and found an insufficient strength over the long-term in the initial high temperature, even though a rapid growth of concrete at the early age was observed. In the low curing temperature of 10 °C, the compression strengths of ordinary and high-performance concrete lessened by 30% and 19%, respectively, compared to the specimens with standard cure (23 °C) [6]. Lothenbach et al. [7] studied the effect of temperature on Portland cement hydration products and found that with the increase of temperature, the formation of calcium silicate hydrate (C-S-H) gel in concrete was denser, but the distribution of hydration products was more uneven, and the porosity was coarser. The curing temperature in the exiting research was generally constant, and few studies paid attention to the changing curing temperature. When the external temperature changes, the temperature stress will be generated in the interior of concrete due to the different thermal expansion coefficients of aggregate and cement paste in concrete [8]. Moreover, when concrete is placed in a long-term environment with large temperature variation, it is easier to form thermal fatigue accumulation, which will cause the formation of internal cracks and affect the safety and service life of the concrete structure.

In recent years, an effective method of adding mineral admixtures was adopted by scholars to improve the performance of concrete [9,10,11]. According to the research of Lee [12] and Johari [13], mineral admixtures (fly ash and slag) can fill voids in concrete and react with cement hydration products to form calcium silicate hydrate, increasing the compaction of concrete, optimizing the pore structure, and improving the ability to resist cracking. Golewski et al. [14] claimed that replacing cement with fly ash can improve the compression strength and fracture toughness of mature concrete, and there is an optimal amount of fly ash of approximately 20%. Uysal et al. [15] used various mineral admixtures to replace cement in self-compacting concrete. The results showed that fly ash and granulated blast furnace slag significantly increased the workability and compression strength of self-compacting concrete mixtures, and the best resistance to sodium and magnesium sulphate attacks was obtained from a combination of 40% granulated blast furnace slag with 60% cement. In addition, the use of mineral admixtures to replace cement in concrete can effectively save engineering costs, reduce the early hydration heat of concrete, and the thermal expansion coefficient of cement slurry, resulting in the reduction of concrete shrinkage and crack formation [16]. Zhang et al. [17] evaluated the effect of mineral admixtures on the hydration heat of the binder paste in high-performance concrete and found that mineral admixtures greatly reduced the hydration heat and the exothermic rate and prolonged the arrival time of the highest temperature, particularly when two or three types of mineral admixtures were added at the same time. Shui et al. [18] investigated the effects of the commonly used mineral admixtures (fly ash, ground granulated blast furnace slag, and silica fume) on the thermal expansion properties of hardened cement pastes (HCP). The result showed replacing Portland cement with mineral admixtures was found to lower the coefficient of thermal expansion of HCP, which was beneficial for mitigating the thermal mismatch between the HCP and the aggregates and reducing the occurrence of cracks in the interface transition zone.

In the process of infrastructure construction in Northwest China, the use of concrete with mineral admixtures may be able to withstand the harsh environment and bring considerable economic benefits. However, there were few studies that paid attention to the characteristics of concrete containing mineral admixtures in such a complex environment. Only Jiang et al. [19] investigated experimentally autogenous shrinkage behaviors of high-performance concrete containing fly ash and blast-furnace slag exposed to different isothermal temperatures and came to the conclusions that slag and fly ash resulted in significant increase and decrease of autogenous shrinkage, respectively. Moreover, the final properties of concrete are largely depended on the initial curing conditions because of the physical and chemical effects during the early hydration process [20]. Therefore, this article focused on the early-age mechanical properties and microstructure of concrete with mineral admixtures. The harsh environment of low humidity and large temperature variation in the northwest of China was simulated by using an environmental chamber. Moreover, concretes with different contents of mineral admixtures were prepared and the actual curing conditions of the concrete were realized in the laboratory. The mechanical characteristics including compression strength and fracture properties of concretes were experimented under different curing ages, and the microstructure of concrete was detected. Basing on the test results, the influence of the harsh environment on the concretes with mineral admixtures were analyzed, and the optimal ratio of mineral admixtures in concrete was proposed. These research results can provide a theoretical basis for the application of concrete with mineral admixture in the environment of low humidity and large temperature variation.

## 2. Materials and Methods

### 2.1. Materials

The Portland cement of P.O. 42.5 (Chinese national standard GB 175-2007 [21]) was used with a specific surface area of 374 m^2^/kg in this study. The fine aggregate utilized was natural river sand with a fineness modulus of 2.85 and an apparent density of 2640 kg/m^3^. The coarse aggregate was natural crushed stone with a particle size of 5−20 mm and an apparent density of 2760 kg/m^3^. The used superplasticizer, working as a water reducing admixture, had a water-reducing rate of 25–30%. The water used in the tests was local tap water.

Two kinds of mineral admixtures were adopted in this research, including fly ash and blast-furnace slag. The fly ash is first level in Class F according to the Chinese national standard GB/T 1596-2017 [22], and the slag is Class S 95 according to the Chinese national standard GB/T 18046-2008 [23]. The detailed properties of the fly ash and slag are listed in Table 1.

### 2.2. Specimens Preparation

In the laboratory trials, four groups of concrete with a strength grade of C 45 were manufactured according to the Chinese national standard JGJ 55-2011 [24], and the mix proportions are given in Table 2. For the ordinary concrete (OC group), the water–cement ratio was 0.4, and the sand ratio was 38%. According to the research results of Li [25], the method of adding fly ash and slag in combination is more beneficial to improving the performance of concrete than adding one of them alone. Moreover, the most appropriate proportion of mineral admixture in concrete was 30−40% by replacing part of the cement in concrete. Therefore, three groups of concrete containing mineral admixtures were prepared in this article, with names of F10S20, F15S15, and F15S20. The numbers after the letters “F” and “S” represents the percentages of fly ash and slag replacing cement, respectively.

**Figure 1:**
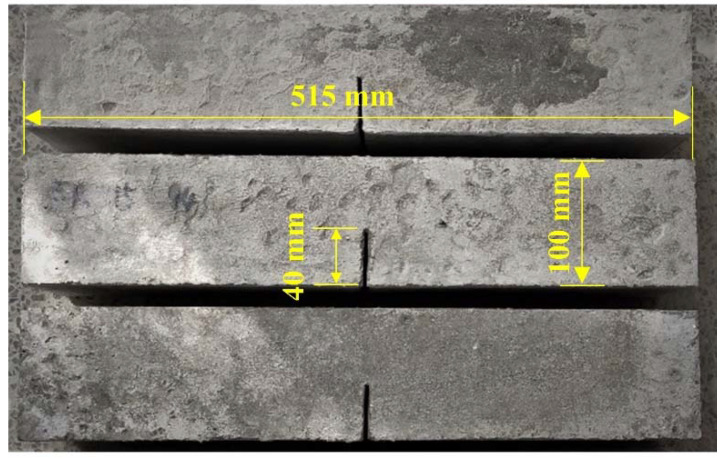
Concrete prisms with pre-notch.

In each group of concrete, six specimens were prepared, including three cube specimens with size of 100 mm × 100 mm × 100 mm for compression strength tests and three prism specimens with size of 100 mm × 100 mm × 515 mm for fracture tests. When the prisms were manufactured, a steel plate with a dimension of 100 mm × 40 mm × 3 mm was welded in the middle of the concrete mold to prefabricate a notch with a width of 3 mm and a depth of 40 mm in the specimens, as shown in Figure 1.

### 2.3. Curing Condition

**Figure 2:**
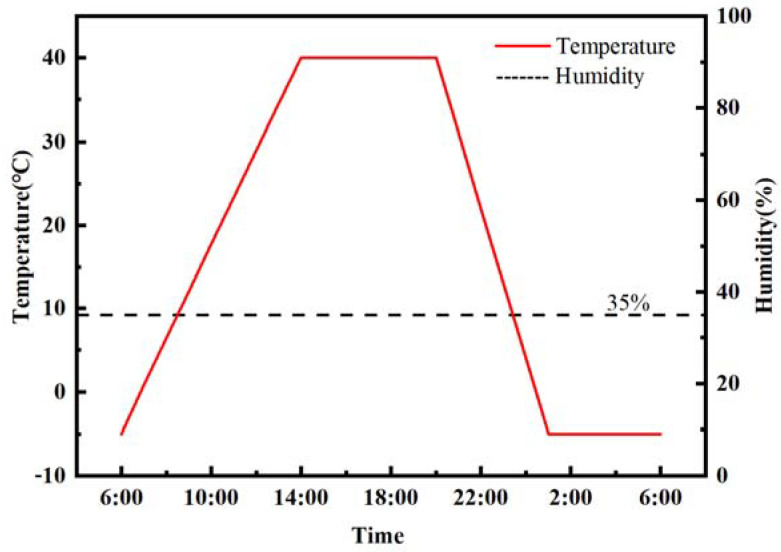
The detailed change process of humidity and temperature in one day in the environmental chamber.

An environmental chamber was utilized to simulate the environment of low humidity and large temperature variation in Northwest China. The meteorological data from 1990 to 2020 were analyzed, and the most adverse environmental state was implemented by the environmental chamber: The humidity was maintained at 35%, and the temperature changed periodically from −5 °C to 40 °C every day. From 1:00 a.m. to 6:00 a.m., it was a constant of −5 °C. Then, the temperature gradually raised to 40 °C from 6:00 a.m. to 14:00 p.m. This high temperature remained until 20:00 p.m. and gradually dropped to −5 °C at 1:00 a.m. the next day. The detailed change process of humidity and temperature in one day was displayed in Figure 2.

After cured for one day under indoor conditions, the concrete specimens were demolded and then placed into the environmental chamber. In order to simulate the real curing condition during construction, a layer of water-saturated geotextile (sprinkling water every day) was covered on the concrete specimens in the first 14 d. Then the geotextile was removed to expose the concrete to the environmental chamber with no sprinkling water from 14 to 28 d.

### 2.4. Experimental Methods

#### 2.4.1. Compression Test

The compression strength tests were conducted according to the Chinese national standard GB/T 50081-2019 [26]. When the concretes reached the curing ages of 3 d, 7 d, 14 d, and 28 d, the cube specimens were taken out from the environmental chamber, and the compression tests were carried out by an electro-hydraulic servo universal testing machine of WAW-2000 (was produced by Shanghai Hualong Test Instruments Corporation, Shanghai, China and has a maximum force range of 2000 KN). During the tests, the loading rate was controlled to be 0.5 MP/s until the peak load. Then the compression strength of each test specimen can be calculated by Equation (1). Note that each group of concrete had three specimens and the compression strength was the average value of the three specimens. Equation (1) is calculated as follows:

(1) $$f_{cc}=\frac{P}{A}$$

where *f_cc_* is the compression strength of the cube specimen (MPa), *P* is the peak load during the compression test (N), and *A* is the bearing area of the specimen (mm^2^).

#### 2.4.2. Fracture Test

**Figure 3:**
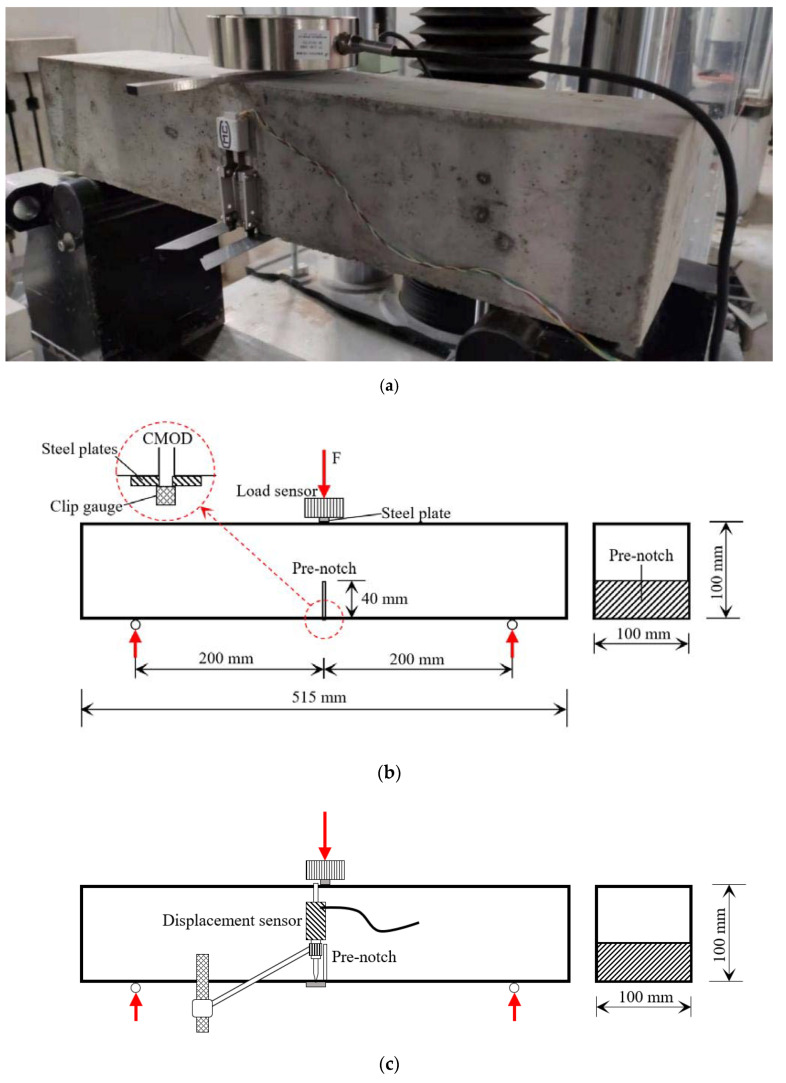
Schematic diagram of three-point bending fracture test: (**a**) experimental setup; (**b**) schematic of front view; (**c**) schematic of rear view.

Due to the harsh environment in the western region of China, concrete is prone to fine cracks during curing. These microcracks will develop, connect, and merge into one or more macrocracks, which can expand gradually and may lead to structural damage. Traditional strength measurements cannot study the effect of cracks on the structure, so the early-age fracture properties of the concretes were detected by a method of three-point bending test at the ages of 3 d, 7 d, 14 d, and 28 d. The tests were carried out by using an electro-hydraulic servo universal testing machine of type WAW-1000 (was produced by Shanghai Hualong Test Instruments Corporation, Shanghai, China and has a maximum force range of 1000 KN), as shown in Figure 3a. A load sensor with a measuring range of 30 KN was utilized to record the load during the tests, under which was a steel plate working for load transferring. A clip gauge was installed at the mouth of the pre-notch to measure the crack mouth opening displacement (CMOD), as shown in Figure 3b. A displacement sensor was used to measure the mid-span deflection of the test specimen, as shown in Figure 3c. During the tests, the loading rate was controlled to be 0.1 mm/min.

After facture tests, the double-K fracture mode [27] was used to describe the fracture propagation, thus three parameters were used to express the fracture properties of concretes: initial fracture toughness, unstable fracture toughness, and fracture energy. The detailed calculation formulas for these three parameters can be founded in our previous literature [28]. Note that three specimens were fractured for each group of concrete, and the average values of the three specimens were adopted for the fracture parameters.

#### 2.4.3. Scanning Electron Microscope (SEM) Observation

To research the influence of mineral admixture on the internal microstructure of concrete under the harsh environment. Scanning electron microscope (SEM) observation for concrete was conducted. After fracture tests, the concrete specimens were cut into cube samples with side length less than 10 mm. The cubes were dried in an oven for 24 h at a temperature of 60 °C and then placed into anhydrous ethanol to prevent further hydration [28]. Before SEM observation tests, the cube samples were re-dried and plated with a gold film. Then the microstructure of the concrete was observed by a scanning electron microscope.

#### 2.4.4. Air-Void System Determination

The internal pore structure of concrete can explain its mechanical properties, so the linear traverse method [29] was used to determine the air-void system of concrete according to the Chinese national standard SL/T 352-2020 [30] by using a hardened concrete pore structure analyzer. The specific steps were as following:

- Cutting the concrete specimens to make an observation surface, which must be perpendicular to the casting surface. The observation surface was smoothened and washed several times until it was flat with only pores and void left. Drying the specimens at a temperature of 105 ± 5 °C before detection.
- Observing the smooth surface by using the hardened concrete pore structure analyzer. The observation area was divided into multiple sub-areas with the same size and each sub-area was taken photos. These photos were binarized for determining the number of air-voids and the chord length by the linear traverse method.
- According to the collected information, the hardened concrete pore structure analyzer calculated the air-void system parameters of concrete, including the air content, average air-void size, and spacing factor. The air content can be considered as the porosity of concrete. The spacing factor represents the average distance between any point in the concrete matrix and the air-void, and a lower value of spacing factor means more air-voids in the observation area.

For each group of concrete, three observation surfaces were prepared, and the air-void system parameters were the average values of the three specimens.

#### 2.4.5. X-ray Diffraction Test

To study the hydration degree of cement in concrete, crumb inside concrete was dried and grounded into power for X-ray diffraction test to detect the phase composition by using an X-ray diffractometer. During the test, the scanning speed was 0.15 s/step, the step length was 0.013 mm, and the scanning angle range is 5–70°.

## 3. Results and Discussion

### 3.1. Mechanical Characteristics

#### 3.1.1. Compression Strength

**Figure 4:**
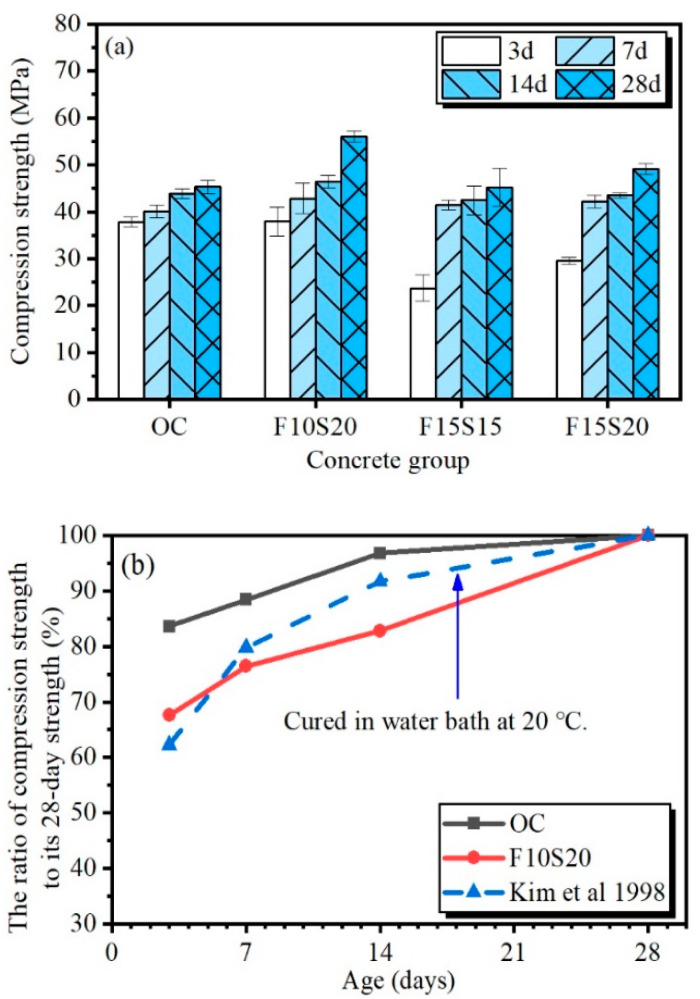
The compression strength of concrete: (**a**) The compression strength of the four types of concrete with curing age; (**b**) The ratio of compression strength to its 28-day values with curing age.

The early-age compression strengths of the four types of concrete were displayed in Figure 4, which all increased with the growth of the curing age. At the age of 3 d, the strength of the OC concrete was higher than that of concretes with mineral admixtures, indicating that the hydration degree of OC concrete was higher before 3 d. This may be because OC concrete had more cement, and cement has higher activity than fly ash and slag in the early days of curing. During the curing time of 3 d to 14 d, the strengths of all the concretes increased gradually, but the growth rates of the concretes with mineral admixtures were faster. At the 14th day, the strengths of F10S20, F15S15, and F15S20 were 105.8%, 96.8%, and 99.2% of that of OC concrete, respectively. At this stage, the effect of mineral admixtures became visible, and the hydration degree of concrete with mineral admixtures developed. On the one hand, the spherical vitreous particles in fly ash can help cement particles to disperse evenly and expand the hydration space of cement, thus promoting hydration reaction [31]. On the other hand, the active SiO_2_ and Al_2_O_3_ in fly ash and slag can react with Ca(OH)_2_ in the presence of water and produced calcium silicate hydrate and calcium sulphoaluminate hydrate, resulting in the consumption of Ca(OH)_2_ and the promotion of the hydration reaction [32]. After 14 d of curing, the strengths of the concretes still increased even though they were exposed to the environment of low humidity and large temperature variation. At the 28th day, the strengths of F10S20, F15S15, and F15S20 concrete were 123.9%, 99.8%, and 108.5% of that of OC concrete, respectively, showing the positive effect of mineral admixtures on the strength of concrete in the harsh environment. This is because, under normal curing conditions, the 28-day strength of mineral admixture concrete is lower than that of ordinary concrete [25]. Moreover, according to the existing research [33], the early-age activity of slag is higher than the fly ash, and the activity of fly ash decreases with its content in concrete. Therefore, the strength of F15S15 concrete was lower than that of F10S20 and F15S20. Comparing with the other two kinds of concretes with mineral admixtures, the F10S20 concrete expressed higher strengths at all measured ages, declaring that this content of mineral admixtures was most beneficial to the concrete strength.

To research the growth rule of the strength of concrete in the environment of low humidity and large temperature variation, the ratios of concrete compression strength to its 28-day strength of OC and F10S20 concrete were plotted in Figure 4b, as well as the concrete under normal curing condition [34]. When the ordinary concrete was cured in water bath at 20 °C, the compression strength increased gradually in the 28 d of curing and the increase rate declined with curing age (the dotted line in Figure 4b). In the environment of low humidity and large temperature variation, the strength of ordinary concrete mainly increased in the first three days, resulting in 3-day strength ratio of 83.6%, which was much higher than the ratio of 62.3% in the water bath at 20 °C. The reason may be the hydration promotion of the external high temperature, which played a more important role than the low temperature in the experimental process. After 14 d, the strength of OC concrete increased slowly, resulting from the negative effect of the harsh environment. For F10S20 concrete, the strength showed a liner increase during the 28 d of curing, indicating that the addition of mineral admixtures had an inhibitory effect on the early strength of concrete, but had a good resistance to the harsh environment after 14 d.

#### 3.1.2. Load-CMOD Curves

**Figure 5:**
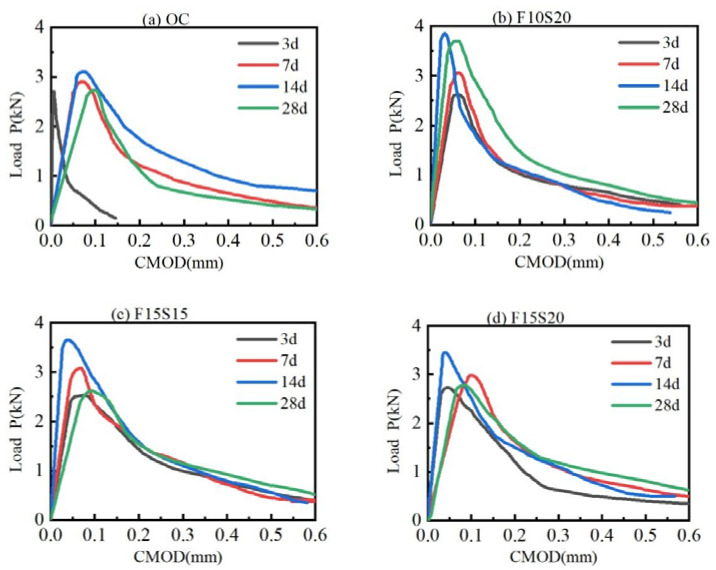
P-CMOD curves of the four types of concrete at different curing ages: (**a**) OC; (**b**) F10S20; (**c**) F15S15; (**d**) F15S20.

The load-CMOD (P-CMOD) curves of the four types of concrete at early ages were plotted in Figure 5. It was seen that the fracture process of all the concretes can be divided into three stages, which were consistent with the research results of Mier [35]. (1) The first is the elastic behavior stage. When loading began, the load increased linearly with CMOD. In this stage, the concrete did not crack. (2) The second is the microcrack stage. As the CMOD grew, the load still increased with the CMOD, but the increase rate gradually slowed down until the maximum load. In this stage, the concrete began to appear microscopic cracks. (3) Third is the macrocrack stage. After the load reached the maximum value, it dropped with the CMOD increased. In this stage, the macrocracks developed to form penetrating cracks in the specimens.

From Figure 5, the peak load of the four kinds of concrete increased firstly and then decreased with the growth of curing age and reached the maximum values at the curing age of 14 d. In the first 14 d, the concretes were covered by saturated geotextiles, so that the wet environment can provide water for the cement hydration. After 14 d, the geotextiles were removed, exposing the concretes to the environment of low humidity and large temperature variation. In this situation, the concretes lost their water supply, and their internal water began to migrate outward because of the humidity gradient, causing the dry shrinkage of the concretes. In addition, the change of external temperature can cause the temperature shrinkage of concretes. These negative effects can bring micro-cracks in the concretes, even though the compressive strengths of the concretes still increased. At the curing age of 3 d, the curve slope of OC concrete in the elastic behavior stage was higher than the other three kinds of concrete with mineral admixtures, indicating that OC concrete had a larger elastic modulus and stronger brittleness in the first 3 d of curing.

#### 3.1.3. Fracture Toughness

**Figure 6:**
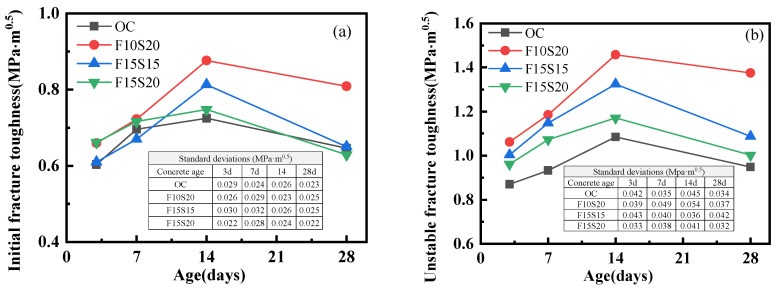
Variation of fracture toughness of concrete with curing age: (**a**) Initial fracture toughness; (**b**) Unstable fracture toughness.

In fracture tests, the initial fracture toughness and unstable fracture toughness represent the ability of concrete to resist cracking and resist crack propagation, respectively. The fracture toughness of concretes versus curing age was shown in Figure 6. The initial toughness and unstable toughness of all kinds of concrete increased firstly and then decreased during the 28 d of curing, which was consistent with the change of the peak load in the P-CMOD curves. This indicated that the fracture properties of concretes were reduced after the specimens were exposed to the harsh environment. However, the fracture toughness of mineral admixtures concrete was higher than OC concrete at almost all measured ages, stating that the addition of fly ash and slag can improve the crack resistance ability of concrete. In the dry environment, the moisture in concrete was easily lost. Nevertheless, the mineral admixtures can fill the micropores in the concrete and effectively block the passage of water. Thus, the water loss of concrete declined, and the dry shrinkage of concrete was reduced [36]. In addition, the cement paste in concrete will shrink more than the coarse aggregate in the environment of large temperature variation due to the difference in thermal expansion coefficients between them, resulting in greater stress on the interfacial transition zone (ITZ) of concrete. In the concretes with mineral admixtures, firstly, fly ash and slag can reduce the primary microcrack in the ITZ and eliminate the stress concentration, improving the tensile strength of this zone. Secondly, the mineral admixtures can improve the strength of ITZ by producing calcium silicate hydrate gel and calcium sulphoaluminate crystals in the pozzolanic reaction [31], which has been proven by the SEM observation results in Section 3.2.1.

Figure 6a displayed the initial fracture toughness of concretes. Before 7 d, the initial fracture toughness of the concretes with mineral admixtures was approximate to OC concrete, and all were higher than OC concrete in the 14th day. At the 28th day, F10S20 concrete had the highest initial fracture toughness, with an increase of 25.2% over OC concrete. Figure 6b showed the unstable fracture toughness of concretes. It can be clearly seen that the unstable toughness of the concretes with mineral admixtures was higher than that of the ordinary concrete in the 28 d of curing. At the age of 28 d, the unstable fracture toughness of F10S20, F15S15, and F15S20 were increased by 45.3%, 15.8%, and 5.3%, respectively, compared to that of OC concrete. Compared with different groups of concretes containing mineral admixtures, F10S20 concrete had the highest fracture toughness, which is consistent with the results of the compression strength, indicating that the addition of 10% fly ash and 20% slag can effectively improve the fracture property of concrete. From Figure 6, the unstable fracture toughness of mineral admixture concrete improved more than the initial fracture toughness compared to ordinary concrete. The reason for this may be that the initial fracture toughness of concrete is closely related to its strength, and the unstable fracture toughness is related to the expansion of its internal microcracks [19]. At the curing age of 28 day, only F10S20 concrete had a significantly higher strength than OC concrete (Figure 4a). Moreover, the internal structure of all the three kinds of mineral admixture concrete can be improved by fly ash and slag.

#### 3.1.4. Fracture Energy

**Figure 7:**
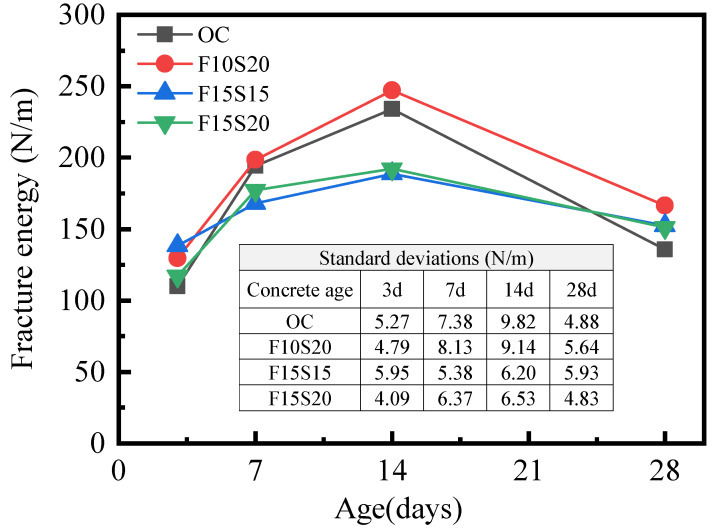
Variation of fracture energy of concrete with curing age.

Fracture energy represents the consumed energy by unit crack area in the process of expansion. The variation of fracture energy of the four types of concretes with curing age was shown in Figure 7. At the curing age of 3 d, the fracture energies of the concretes with mineral admixtures were slightly higher than the OC concrete, resulting from fly ash and slag filling the pores and improving the interface transition zone (ITZ) of the concrete [32]. After 3 d, the fracture energies of all concretes continued to increase. At the curing age of 14 d, the relationship of the fracture energy of the four types of concrete was as follows: F10S20 > OC > F15S20 > F15S15, which is consistent with the compression strength of concrete. After 14 d, the fracture energy of concrete dropped because of the negative effect of the harsh environment, and OC concrete had the largest decline, explaining that the mineral admixtures improved the ability of concrete to withstand dry and large temperature variation. Among the three types of concretes containing mineral admixtures, F10S20 concrete had higher fracture energies than the OC concrete at all measured ages (17.8% at 3 d, 2.0% at 7 d, 5.6% at 14 d, and 22.6% at 28 d), proving that the contents of fly ash and slag in F10S20 concrete were the best in this research.

**Figure 8:**
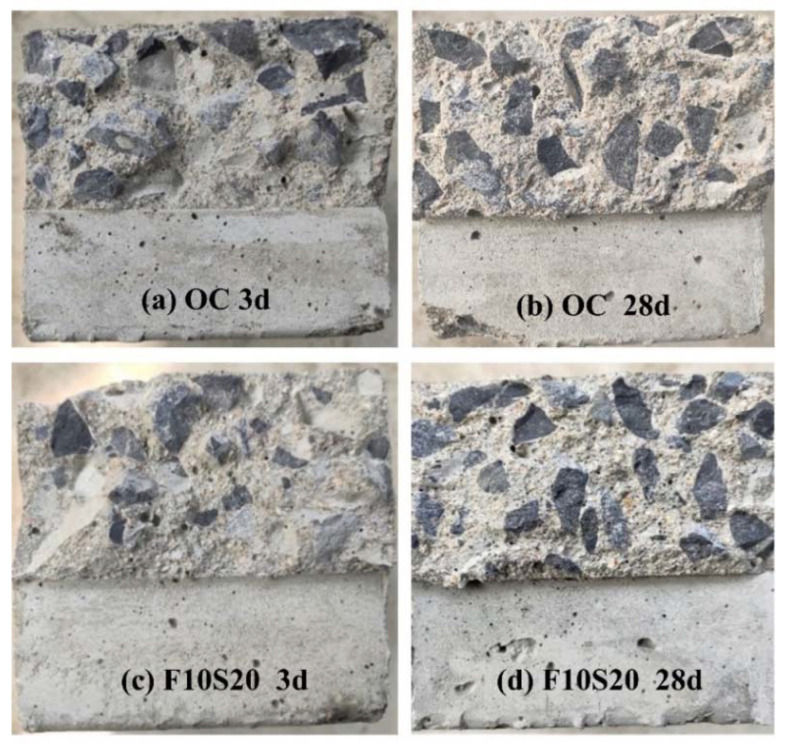
Fracture surfaces of OC and F10S20 concrete specimens: (**a**) OC concrete at 3 d; (**b**) OC concrete at 28 d; (**c**) F10S20 concrete at 3 d; (**d**) F10S20 concrete at 28 d.

The interface transition zone is the weakest and most sensitive area inside the concrete, and the breakdown of concrete often starts here [10]. In the early stage of curing, the hydration degree of cement was low in concrete, so the bonding between the cement paste and the coarse aggregate was weak. When the concrete was fractured, the cracks mainly propagated along the interface transition zone, and the aggregate was pulled out of the cement paste, as shown in Figure 8a,c. With the growth of age, the interface transition zone became strong because of more and more hydration products accumulated in this zone, resulting in the improvement of the bonding capacity of cement paste and aggregate. In this situation, more and more coarse aggregates were destroyed when the concrete fractured, as shown in Figure 8b,d. It was observed that more coarse aggregates were fractured in F10S20 concrete, compared with OC concrete, which also proved that F10S20 concrete had better crack resistance.

From the experimental results of mechanical characteristics, F10S20 concrete revealed the best performance among the three types of concrete with mineral admixtures. One of the reasons for this was that F10S20 concrete had higher content of slag and lower content of fly ash. Slag with high contents of calcium oxide can participate in the hydration process of cement at the early age and improve the hydration products in concrete [37]. The activity of fly ash was low at the early age and high content of fly ash was detrimental to its activity [33]. Therefore, in the environment of low humidity and large temperature variation, 10% content of fly ash with 20% content of slag was an appropriate mixing in concrete in this research. Next, the microstructures of F10S20 concrete were researched to explain its superior performance.

### 3.2. Microstructures of Concretes

#### 3.2.1. SEM Images

**Figure 9:**
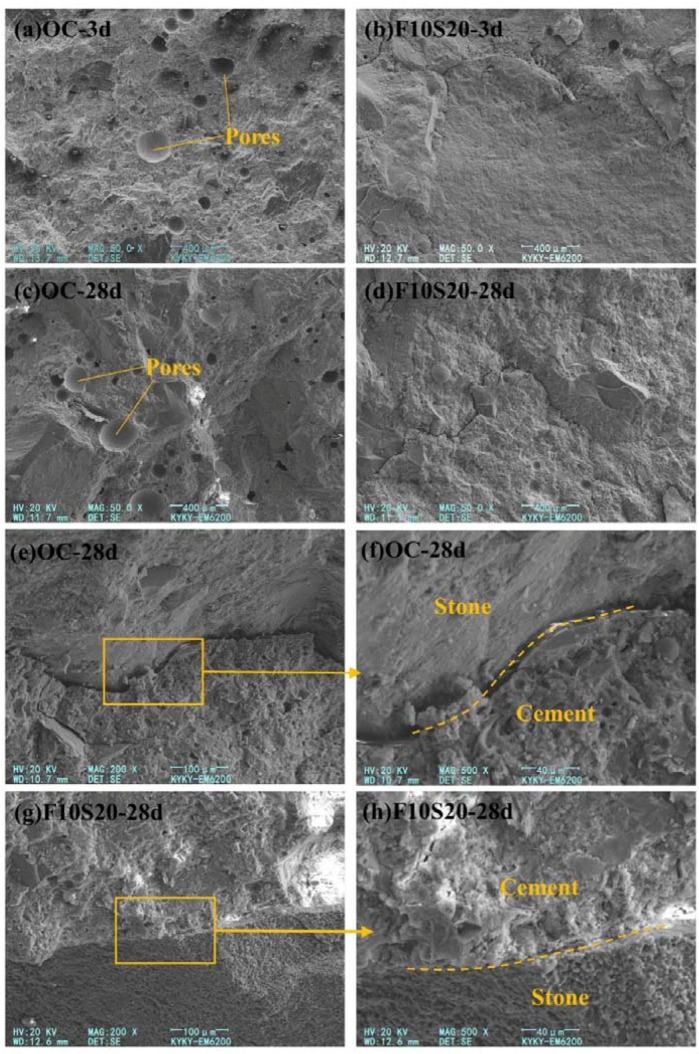
SEM images of OC and F10S20 concrete: (**a**) Microstructure of OC concrete at 3 d; (**b**) Microstructure of F10S20 concrete at 3 d; (**c**) Microstructure of OC concrete at 28 d; (**d**) Microstructure of F10S20 concrete at 28 d; (**e**) ITZ of OC concrete at 28 d; (**f**) Magnified ITZ of OC concrete at 28 d; (**g**) ITZ of F10S20 concrete at 28 d; (**h**) Magnified ITZ of F10S20 concrete at 28 d.

From the SEM images of OC and F10S20 concrete, the influence of mineral admixture on the internal microstructure of concrete can be clearly observed, as shown in Figure 9. From Figure 9a,c, there were many pores with diameter greater than 100 μm in OC concrete at the curing age of 3 d and 28 d, indicating that the compactness of OC concrete remained poor throughout the curing period. Under the same magnification, the pores in F10S20 can hardly be seen in the SEM images, as shown in Figure 9b,d. The comparation of the interfacial transition zone (ITZ) between OC and F10S20 concrete was exhibited in Figure 9e–h. It can be seen that large cracks appeared in the ITZ of OC concrete, indicating that the internal structure of OC concrete has been damaged by the harsh external environment. On the one hand, the water inside the concrete migrated outside in the external dry environment, reducing the hydration products and decreasing the compaction degree in the ITZ, thus resulting in the increasing porosity in the ITZ. On the other hand, due to the different thermal expansion coefficient between cement and stone, the drastic change of external temperature caused them to expand and contract in different degrees, resulting in cracks between them. Compared with OC concrete, the ITZ of F10S20 concrete was denser, which revealed the internal reason why the mechanical properties of F10S20 concrete were better than OC concrete.

#### 3.2.2. Air-Void Characteristics

**Figure 10:**
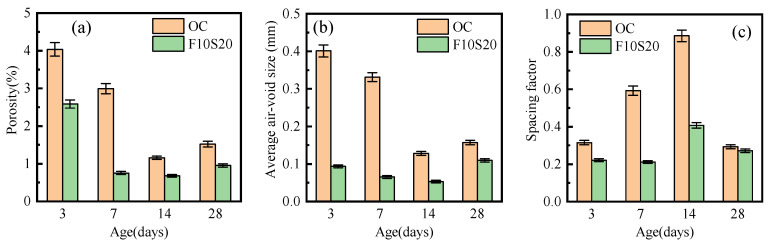
Concrete air-void parameters with curing age: (**a**) Porosity; (**b**) Average air-void size; (**c**) Spacing factor.

To investigate the influence of low humidity and large temperature variation on the concretes, three air-void parameters of OC and F10S20 concrete versus curing age were detected, including porosity, average air-void size, and spacing factor, as shown in Figure 10. The porosity and average air-void size of OC and F10S20 concrete decreased gradually during the first 14 d of curing but increased again in the last 14 d. Before 14 d, the concretes were covered by wet geotextiles, during which the hydration reaction inside the concretes continued, with the number of pores reduced and the pore size becoming smaller [38]. After 14 d, concretes were exposed to the harsh environment, resulting in the loss of the internal moisture. In addition, under the effect of the external temperature variation, internal microcracks appeared in the concrete, decreasing its compaction. Compared with the ordinary concrete, F10S20 concrete had lower porosity and smaller average air-void size at all ages, resulting from mineral admixtures filling the pores and promoting the hydration reaction inside the concrete, which have been confirmed by the SEM observation. The spacing factor represented the average distance between any point in the concrete matrix and the pores. The greater its value, the fewer pores in the observed area. Figure 10c displayed the spacing factors of OC and F10S20 concrete. It can be seen that the spacing factor of OC was higher than F10S20 concrete, explaining that there were fewer pores in OC concrete. However, because of the larger pore size of OC concrete, its porosity was greater than F10S20 concrete.

**Figure 11:**
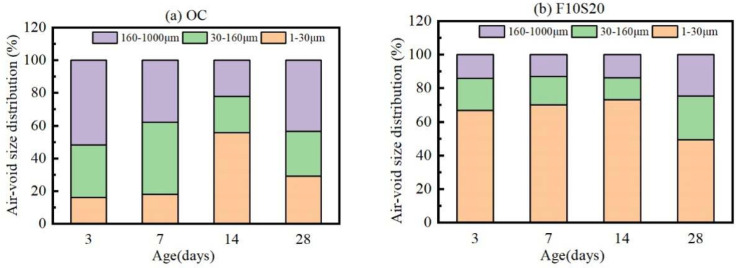
Air-void distribution of (**a**) OC and (**b**) F10S20 concrete at different ages.

The air-void size distribution of OC and F10S20 concrete at different ages was drawn in Figure 11. At the curing age of 3 d, the percentages of air-voids with diameters of 160–1000 μm, 30–160 μm, and 1–30 μm were 51.8%, 31.9%, and 16.3%, respectively. As the curing age grew before 14 d, the percentage of the large pores gradually decreased, while the percentage of small pores gradually increased due to the continuous hydration reaction in the concrete. At the 14th day, the percentages of air-voids with diameters of 160–1000 μm and 1–30 μm were 22.2% and 55.7%, respectively. After 14 d, the percentage of large air-voids increased again and reached 43.5% at the curing age of 28 d, resulting from the internal pores becoming connected under the action of drying shrinkage and temperature shrinkage after the concrete was exposed to the environment of low humidity and large temperature variation. For F10S20 concrete, the percentage of small air-voids with a diameter of 1–30 μm was maintained at a high level, and the number of large air-voids was low in the first 14 d. This declared that the mineral admixtures could improve the pore size distribution in concrete. After 14 d, the percentage of large air-voids with a diameter of 160–1000 μm (24.7% at the 28th day) also increased under the harsh environment, but it was still lower than OC concrete. Note that, due to the limitation of the measurement method adopted in this article, the air-voids with a diameter less than 1 μm could not be distinguished, but the improvement effect of the addition of fly ash and slag on the pore structure of concrete could still be observed.

#### 3.2.3. X-ray Diffraction Analysis

**Figure 12:**
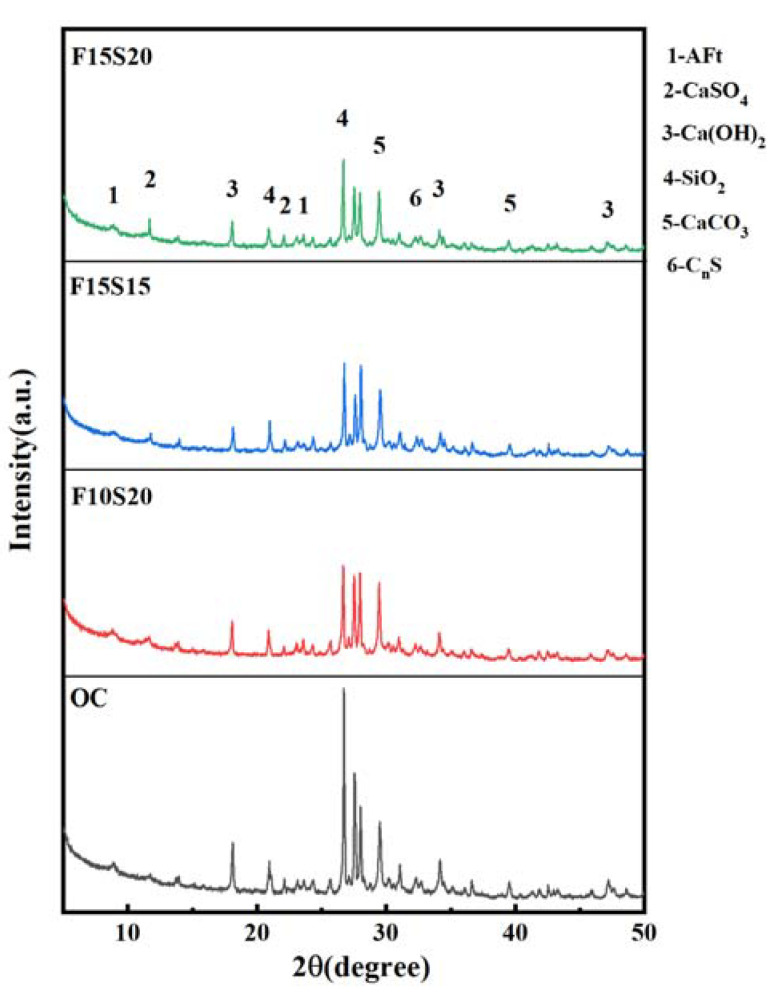
X-ray diffraction pattern of hydration products in concretes at the curing age of 28 d.

X-ray diffraction can be used to analyze the phase composition in concrete, through which the hydration characteristics of concrete can be studied. Figure 12 showed the X-ray diffraction pattern of hydration products in the 4 types of concrete at the curing time of 28 d. It can be seen that there were ettringite (AFt), gypsum (CaSO_4_), calcium hydroxide (Ca(OH)_2_), silica (SiO_2_), calcium carbonate (CaCO_3_), and calcium silicate (C_n_S) in concrete, and SiO_2_ had the highest diffraction peak. This was because the test sample contained a certain amount of sand, which was mainly composed of silica. Ca(OH)_2_, as the hydration product, can reflect the hydration degree in concrete. From Figure 12, the order of the diffraction peaks of Ca(OH)_2_ in the four types of concrete was as follows: OC > F10S20 > F15S20 > F15S15. OC concrete had the highest cement content in the four kinds of concrete, resulting in the highest amount of Ca(OH)_2_ at the curing age of 28 d. Slag contained more CaO, which can generate Ca(OH)_2_ with water. Therefore, F10S20 and F15S20 concrete, which had higher slag content, had higher Ca(OH)_2_ content than F15S15. Moreover, the diffraction peaks of CaSO_4_ (2θ = 11.67°) in the concrete with mineral admixtures was higher than that in OC concrete, indicating that more CaSO_4_ was produced in concrete because of the addition of fly ash and slag. The produced CaSO_4_ and AFt can both fill in the pores and improve the compaction of concrete.

## 4. Conclusions

This paper investigated the early-age mechanical characteristics and microstructures of concrete with mineral admixtures under the environment of low humidity and large temperature variation. Four types of concrete, including ordinary concrete and three kinds of mineral admixtures concrete, were prepared and cured in an environmental chamber, which was used to simulate the real curing condition during construction in Northwest China. The compression strength, fracture properties, SEM images, air-void characteristics, and X-ray diffraction features of concrete were researched. The following conclusions can be drawn.

Under the simulative curing environment during construction, the compression strength of ordinary concrete was higher before the first 3 d but was surpassed by the concrete with mineral admixtures after exposure to the curing environment of low humidity and large temperature variation. The fracture properties of concrete increased in the first 14 d and decreased in the last 14 d during the curing period, and the fracture toughness of the mineral admixture concrete was higher than the ordinary concrete. Among the three kinds of concrete containing mineral admixtures, F10S20 concrete showed the best performance, whose compression strength, initial fracture toughness, unstable fracture toughness, and fracture energy were 23.9%, 25.2%, 45.3%, and 22.6% higher than OC concrete, respectively, at the curing age of 28 d.

The addition of fly ash and slag can weaken the negative effects of the harsh environment of low humidity and large temperature variation on concrete microstructure and cement hydration. Firstly, the mineral admixture can reduce the pores in the concrete caused by drying shrinkage and temperature variation and can reduce the size of the pores. Secondly, fly ash and slag can improve the hydration degree in concrete, even under the condition of lacking water.

Even though the experimental design in this research was specific, including adopting the environmental parameters of Northwest China and few kinds of concrete with mineral admixtures being prepared, the research results can still provide valuable theoretical guidance for the application of mineral admixtures concrete in the environment of low humidity and large temperature variation.

## Figures and Tables

**Table 1:** The properties of the mineral admixtures.

**Fly Ash**	**Fineness** **(%)**	**Water Demand Ratio (%)**	**Ignition Loss** **(%)**	**SO_3_ Content (%)**	**Density** **(g/cm3)**	**Water Content (%)**
	10.8	91.5	4.6	2.2	2.3	0.5
**Blast-Furnace Slag**	**Specific Surface Area** **(m^2^/kg)**	**Flow Ratio** **(%)**	**Activity Index 28d** **(%)**	**Density** **(g/cm^3^)**	**Ignition Loss** **(%)**	**Water Content** **(%)**
	429	98.0	98.5	3.1	1.24	0.25

**Table 2:** Concrete mix proportions (kg/m^3^).

Groups	Water	Cement	Fly Ash	Slag	Sand	Stone	Superplasticizer
OC	160	400	0	0	699	1141	1.2
F10S20	160	280	40	80	699	1141	1.2
F15S15	160	280	60	60	699	1141	1.2
F15S20	160	260	60	80	699	1141	1.2

## Data Availability

The data presented in this study are available on request from the corresponding author. The data are not publicly available due to privacy.
